# Effect of exercise training on cardiac autonomic function in type 2 diabetes mellitus: a systematic review and meta-analysis

**DOI:** 10.1186/s13643-025-02772-9

**Published:** 2025-02-04

**Authors:** Sohini Raje, G Arun Maiya, Padmakumar R, Mukund A. Prabhu, Krishnananda Nayak, Shivashankara KN, BA Shastry, Megha Nataraj

**Affiliations:** 1 Centre for Podiatry & Diabetic Foot Care and Research, Department of Physiotherapy, Manipal College of Health Professions, Manipal Academy of Higher Education, Manipal, Karnataka 576104 India; 2 Department of Cardiology, Kasturba Medical College-Manipal, Manipal Academy of Higher Education, Manipal, Karnataka 576104 India; 3 Department of Cardiovascular Technology, Manipal College of Health Professions, Manipal Academy of Higher Education, Manipal, Karnataka 576104 India; 4 Department of Medicine, Kasturba Medical College-Manipal, Manipal Academy of Higher Education, Manipal, Karnataka 576104 India; 5 Department of Cardiovascular & Respiratory Physiotherapy, MGM College of Physiotherapy, Navi Mumbai, Maharashtra 400705 India

**Keywords:** Type 2 diabetes, Cardiac autonomic function, Exercise, Cardiac autonomic neuropathy, Meta-analysis, Lifestyle management

## Abstract

**Background:**

Cardiac autonomic neuropathy (CAN) is an underdiagnosed complication of type 2 diabetes mellitus (T2DM) and is associated with cardiovascular morbidity and mortality. Cardiac autonomic reflex tests (CARTs) are the gold standard; they are non-invasive and clinically feasible for screening CAN. The objective of the present meta-analysis was to examine exercise’s effect on cardiac autonomic function using CARTs in T2DM.

**Methods:**

The Preferred Reporting Items for Systematic Review and Meta-Analysis Checklist (PRISMA) was used. Electronic databases were systematically used to retrieve relevant studies after title and abstract screening. Studies utilizing exercise training with cardiac autonomic function (CARTs) outcomes in individuals with type 2 diabetes mellitus were included. The meta-analysis was conducted using RevMan 5.4.1, using the random effects model, and appropriate tests for heterogeneity. The Cohrane ROB-2 tool was used for randomized controlled trials (RCTs) and the ROBINS-I tool for non-RCT for risk of bias assessment were used.

**Results:**

Three studies were included (two for meta-analysis), considering the outcome of the E:I ratio, 30:15 ratio, and Valsalva ratio. The studies did not show any influence on the E:I and 30:15 ratio in the pooled analysis with a low risk of ineffectiveness for the exercise intervention. Exercise training significantly affected the Valsalva ratio. A different type of exercise intervention was utilized in all three studies. There was a low to moderate certainty for the evidence.

**Conclusion:**

The results indicate that further robust and high-quality randomized controlled trials utilizing cardiac autonomic reflex tests (which have clinical and physiological relevance) in type 2 diabetes mellitus are required for drawing conclusions.

**Systematic review registration:**

PROSPERO CRD42023445561.

**Supplementary Information:**

The online version contains supplementary material available at 10.1186/s13643-025-02772-9.

## Background

Diabetes mellitus (DM) is an ongoing health emergency of the twenty-first century, with the age-standardized rate of mortality reported to have increased by 13% from 2000 to 2019 in low-middle-income countries [1]. The prevalence of diabetes mellitus is 21.4% in India, with 90% of individuals with DM having type 2 diabetes mellitus (T2DM) [2, 3]. Cardiac autonomic neuropathy (CAN) is an underdiagnosed complication of T2DM associated with mortality and morbidity [4]. CAN is a strong predictor of silent myocardial ischemia, estimated glomerular filtration rate decline, anemia, ischemic stroke, diabetic nephropathy, and left ventricular diastolic dysfunction [5–9]. The prevalence of CAN in T2DM is reported between 15.3 and 68% [10–14]. The Indian prevalence was said to be slightly higher, between 70 and 85% [15, 16]. The wide variation in prevalence is due to the different characteristics of the population, diagnostic criteria, and definitions of CAN [17].

The management of T2DM is by lifestyle modification, including exercise, diet, and counseling along with pharmacological management. Exercise reduces insulin resistance, HbA1C, triglycerides, blood pressure, fat mass, strength, and lean body mass in T2DM [18]. Glycemic control is currently the primary tool for reversing or preventing CAN in T2DM [19].

Cardiac autonomic function is screened using cardiac autonomic reflex tests (CARTs), heart rate variability (HRV), and baroreflex sensitivity (BRS) [8]. Although HRV is an objective, non-invasive measure of autonomic function, it is over-utilized and has shortcomings. One of the reasons for limitation is the oversimplification of the relationship between the sympathetic and parasympathetic nervous systems and the frequency component of HRV [20]. Baroreflex sensitivity (BRS) is “the change in the interbeat interval in milliseconds per unit change in blood pressure” [21]. BRS is performed by pharmacological methods (that is, by intravenous administration of a vasoconstrictor drug), the Valsalva maneuver, and the neck chamber technique (non-invasive but used in research settings) [22]. Thus, the need to utilize other more accessible and non-invasive tests, such as CARTs, becomes essential. CARTs have a high sensitivity and specificity (80–90%) for cardiac autonomic neuropathy and utilize heart rate and blood pressure using a battery of 5 simple tests proposed by Ewing [23, 24].

Previous systematic reviews examined the effect of exercise training on HRV, BRS, and heart rate recovery as a measure of cardiac autonomic function in T2DM [25–29]. They reported that exercise aids in inhibiting sympathetic overactivity and improving parasympathetic function in T2DM, thus improving sympathovagal balance. The present systematic review examines exercise’s effect on cardiac autonomic function using cardiac autonomic reflex tests in individuals with T2DM.

## Methods

The Preferred Reporting Items for Systematic Review and Meta-Analysis Checklist (PRISMA) was used to conduct the systematic review [30]. The checklist can be referred to from the Additional file 1. The review protocol was registered with PROSPERO with the number CRD42023445561.

### Information sources

The searches were done on the following databases by two reviewers (SR and GAM): PubMed, Embase, Web of Science, Ovid MEDLINE, SCOPUS, and ProQuest. The references of the included studies were also searched for relevant articles.

### Keywords and MeSH terms

The keywords searched were as follows: “cardiac autonomic function,” “cardiac autonomic neuropathy,” “cardiac autonomic reflex tests,” “parasympathetic function,” “sympathetic function,” “type 2 diabetes,” “exercise,” “physical activity” with BOOLEAN operators AND, OR. No other filters were applied. The search was performed from 17 August 2023 to 24 August 2023. The detailed search strategies are shown in Additional file 2.

### Study selection

The citations from the searches were imported to Rayyan software. The study selection process was divided into 2 phases—(1) title and abstract screening and (2) full-text screening by two reviewers (SR and GAM), and any conflicts were resolved by a third reviewer (MN).

### Eligibility criteria

#### Population

- The studies with individuals over 18 years of age, of any gender, with physician-diagnosed T2DM, managed by pharmacological (oral hypoglycemics, insulin, or both) and non-pharmacological management (diet, counseling)
- Studies were excluded when participants were diagnosed with type 1 diabetes mellitus, gestational diabetes mellitus, or other genetic forms

#### Intervention

- Studies were included if the intervention provided was exercise or physical activity. The intervention should be structured using the FITT principle (Frequency, Intensity, Time, Type), supervised, unsupervised, or both—exercise in the form of aerobic, resistance, combined, moderate-intensity continuous, or high-intensity interval training
- Studies were excluded if they involved interventions such as yoga, breathing exercises, tai chi, diet only, and acupuncture

#### Control

- Studies were included if the control group participants were provided with medical management only or any other exercise or non-exercise-based intervention

#### Outcome

- Studies with the outcome of cardiac autonomic reflex tests included resting heart rate, average deep breathing difference, respiratory sinus arrhythmia index, expiratory: inspiratory ratio, 30:15 ratio, and Valsalva ratio
- Studies that exclusively performed other methods of cardiac autonomic function testing (HRV, BRS, heart rate recovery) were excluded

### Types of studies

Randomized controlled trials (RCTs), non-randomized controlled trials (non-RCTs), and pre-post studies were included in the review. Conference abstracts, case series, case reports, editorials, qualitative studies, commentaries, short communications/correspondence, research briefs, or letters to editors were excluded.

### Data extraction

A customized data extraction form was used to extract data from the studies. Two reviewers performed the data extraction process. The following was extracted from the studies—age, gender, type of study, sample size, details of the intervention (FITT), and results (mean difference, mean, standard deviation, median, interquartile range) of the cardiac autonomic reflex test outcomes. If any required data was unavailable, the authors were contacted through email for the relevant information.

### Risk of bias (ROB) of included studies

The Risk Of Bias In Non-randomized Studies—of Interventions (ROBINS-I) was used for ROB assessment of non-RCTs [31]. The ROBINS-I examines the ROB based on the bias in non-RCTs based on confounding, selection of participants, deviation from intended interventions, missing data, and measurement of outcomes. The Cochrane ROB 2 tool was used for the RCT for the outcomes of E:I ratio, Valsalva ratio, and 30:15 ratio [32]. Two reviewers performed the ROB assessment independently. The third reviewer resolved any discrepancies in the ROB assessment between the other two reviewers.

### GRADE assessment

The certainty of the evidence was evaluated using the GRADEpro GDT software [33]. The GRADE approach uses components such as the risk of bias, inconsistency, indirectness, and imprecision, along with other considerations, to evaluate the certainty of evidence in terms of low, moderate, and high.

### Therapeutic quality of exercise program

The international Consensus on Therapeutic Exercise aNd Training (i-CONTENT) tool was used to examine the therapeutic quality of the exercise program [34]. The tool aids in the transparent assessment of the exercise program to identify the heterogeneity of the exercise intervention. It consists of 7 items—(1) Patient selection, (2) Dosage of exercise program, (3) Type of exercise program, (4) Presence of a qualified supervisor, (5) Type and timing of outcome assessment, (6) Safety of the exercise program, (7) Adherence to the exercise program. Two reviewers performed the evaluation. The third reviewer resolved any disagreements in the responses.

### Data synthesis

The data extracted was entered in Microsoft Excel. Reporting of the cardiac autonomic reflex test outcomes was based on the normality of the data, that is mean, standard deviation, median, interquartile range, and mean difference for the cardiac autonomic reflex test variables. All outcomes were reported in mean and standard deviation for the included studies, except for the outcome of change in systolic blood pressure in one study. The variables of cardiac autonomic reflex tests were in natural log-transformed value in one study [35]. These were transformed into raw scores of mean and standard deviations based on a formula [36]. Meta-analysis was performed using RevMan 5.4.1 [37], and the effect sizes for the outcomes were analyzed, computed, and calculated for the cardiac autonomic reflex test parameters. The fixed effects model was utilized. Heterogeneity was checked using forest plots, chi-square test, and *I*^2^ value. *I*^2^ < 25% was considered low heterogeneity, 25% to 50% was considered modest heterogeneity, and > 50% was regarded as high heterogeneity. Funnel plots were used to check for publication bias.

## Results

**Fig. 1:**
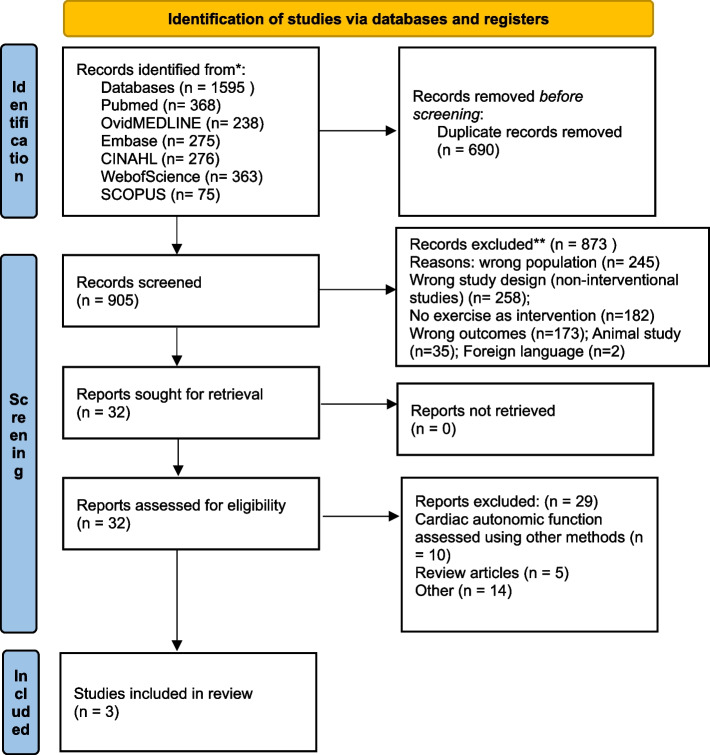
PRISMA flowchart

One thousand five hundred ninety-five articles were retrieved through electronic databases- PubMed, Ovid MEDLINE, Embase, CINAHL, Web of Science, and SCOPUS. After removing duplicates, 905 records underwent title and abstract screening, of which 32 records underwent full-text screening, and three studies were included for the review and two [35, 38] the meta-analysis. Figure 1 shows the PRISMA flow diagram.

### ROB of included studies

The ROB of the non-RCTs [35, 38] was performed using the ROBINS-I tool, which showed a low risk of bias for most of the domains. The RCT [39] showed some concerns with respect to the outcome of the E:I ratio and the 30:15 ratio. This was mainly due to the ROB arising from the randomization process, the effect of assignment to intervention, and the selection of the reported result. The Valsalva ratio showed an overall high ROB due to the randomization process, effect of assignment to intervention, measurement of the outcome, and selection of the reported result. The ROB in the included studies is shown in Table 1.

**Table 1:** Risk of bias of included studies

**Study**	**ROBINS-I**							
	**Bias due to confounding**	**Bias in selection of participants into the study**	**Bias in classification of interventions**	**Bias due to deviations from intended interventions**	**Bias due to missing data**	**Bias in measurement of outcomes**	**Bias in selection of the reported result**	**Overall bias**
Sacre et al., 2014 [35]	Low	Low	Low	Low	Low	Moderate	Low	Low
Bonhof et al., 2022 [38]	Low	Low	Low	Low	Low	Low	Low	Low
**Study (outcome)**	**Cochrane ROB-2**							
	**ROB arising from the randomization process**	**Effect of assignment to intervention**	**Effect of adhering to intervention**	**Missing outcome data**	**Risk of bias in measurement of the outcome**	**Risk of bias in selection of the reported result**	**Overall bias**	
Bhati et al., 2023 [39] (E:I ratio)	Some concerns	High	Low	Low	Low	Some concerns	Some concerns	
Bhati et al., 2023 [39] (Valsalva ratio)	Some concerns	High	Low	Low	Some concerns^a^	Some concerns	High	
Bhati et al., 2023 [39] (30:15)	Some concerns	High	Low	Low	Low	Some concerns	Some concerns	

^a^Due to the effort-dependent nature of the Valsalva maneuver

### Study design

The current review included three studies. One was an RCT [39], and two were non-RCTs [35, 38]. Table 2 shows the characteristics of the included studies.

**Table 2:** Characteristics of included studies

Study, country, design	Age (years)		Sample size (*n*)		Exercise intervention					CARTS outcome
	Exercise	Control	Exercise	Control	Frequency (days per week)	Intensity	Time, duration	Type	Supervision (yes/no)	
Sacre et al., 2014 [35] (Australia) Non-RCT	59 ± 10	60 ± 9	29	25	2	Moderate to vigorous (based on RPE and HR monitors)	20 to 40 min, for 12 weeks	Aerobic + resistance	Yes	-E:I ratio, Valsalva ratio, 30:15 ratio -Cessation of anti-hypertensive medication (24 h), smoking, caffeine, alcohol (12 h), and heavy exercise (24 h) -A quiet temperate room with 15-min supine -As per standard protocol
Bonhof et al., 2022 [38] (Germany) Non- RCT	56 (53, 63)^a^	57 (53, 60)^a^	20	23	3	90% of HR_max_and 70% of HR_max_	35 min, for 12 weeks	HIIT	Yes	-E:I ratio, Valsalva ratio, 30:15 ratio -As per standard protocol
Bhati et al., 2023 [39] (India) Parallel arm, single blinded RCT	52.8 ± 6.8	54.0 ± 8.18	28	28	3	Moderate. 65 to 75% of 1RM	60 min, for 12 weeks	Progressive resistance	Yes	-E:I ratio, Valsalva ratio, ∆HR deep breathing, ∆DBP, 30:15 ratio, ∆SBP (orthostatic test) -ANS modifying medications ceased. 9 am to 12 pm -As per standard protocol

*CARTs*Cardiac autonomic reflex test, *RCT*Randomized controlled trial, *RPE*Rating of Perceived Exertion Scale, *HR*Heart rate, *HR*_*max*_Maximum heart rate, *1RM*1 repetition maximum, *HIIT*High-intensity interval training, *E:I*Expiration to inspiration ratio, *DBP*Diastolic blood pressure, *SBP*Systolic blood pressure

^a^Median (interquartile range)

### Participants

The total number of participants was 153, with 77 in the intervention (exercise) group and 76 in the control group. Most participants were reportedly on metformin, sulphonylureas, statins, insulin, beta-blockers, and calcium channel blockers. The demographic details are mentioned in Table 2. Participants who were diagnosed with T2DM, as per American Diabetes Association (ADA) criteria, were included in two studies [38, 39]. One study had participants with T2DM with early and definite cardiac autonomic neuropathy (as per Ewing’s criteria) [39]. Sacre et al. reported participants with cardiac autonomic neuropathy at baseline; individuals had been diagnosed with T2DM with subclinical diastolic dysfunction [35]. All studies excluded participants with underlying cardiovascular disease, morbid obesity, psychiatric illness, or any other condition adversely affecting cardiac autonomic function [35, 38, 39].

### Description of intervention

Table 2 shows the intervention provided to the participants in both groups (exercise group and control group).

#### Frequency

One study [35] reported the frequency of exercise training twice weekly, while two studies reported a frequency of 3 times per week [38, 39].

#### Intensity

Sacre et al. provided moderate to vigorous intensity training using the Rating of Perceived Exertion (RPE) scale [35]. Bhati et al. provided moderate-intensity training based on one repetition maximum (1RM) [39]. Bonhof et al. utilized a high-intensity interval training program based on the maximum heart rate [38].

#### Time

The included studies reported time between 20 min to 60 min per session. The total duration of the intervention was 12 weeks [38, 39] to 6 months [35].

#### Type

One study reported a combined aerobic and resistance exercise program [35]. Bonhof et al. utilized high-intensity interval training, and Bhati et al. used a resistance training program [38, 39].

#### Supervision

Sacre et al. included both supervised and home-based exercise sessions [35]. Bonhof et al. and Bhati et al. reported the intervention to be supervised only at the center [38, 39].

### Therapeutic quality of the exercise program

All studies showed a low risk for ineffectiveness for patient selection, dosage, type of exercise program, and type and timing of outcome assessment. Sacre et al. demonstrated a low risk for ineffectiveness in adherence to the exercise program [35], while the other two studies did not describe adherence [38, 39]. None of the three studies provided details about the safety of the exercise program. Table 3 shows the therapeutic quality of the exercise program.

**Table 3:** Therapeutic quality of exercise program (i-CONTENT tool)

Study	Patient selection	Dosage	Type	Qualified supervisor	Type and timing of outcome assessment	Safety	Adherence
Sacre et al., 2014 [35]	Low risk for ineffectiveness	Low risk for ineffectiveness	Low risk for ineffectiveness	Low risk for ineffectiveness	Low risk for ineffectiveness	No details (probably done)	Low risk for ineffectiveness
Bonhof et al., 2022 [38]	Low risk for ineffectiveness	Low risk for ineffectiveness	Low risk for ineffectiveness	No details (probably done)	Low risk for ineffectiveness	No details (probably done)	No details (probably done)
Bhati et al., 2023 [39]	Low risk for ineffectiveness	Low risk for ineffectiveness	Low risk for ineffectiveness	Low risk for ineffectiveness	Low risk for ineffectiveness	No details (probably done)	No details (probably done)

### Effect of exercise on cardiac autonomic function in the included studies

Sacre et al. reported no statistically significant change in the Valsalva ratio, 30:15 ratio, and the change in systolic blood pressure after standing after 6 months of combined (aerobic and resistance) training [35]. The change in systolic blood pressure improved in the intervention group only (− 13.9 ± 11.6 to − 9.35 ± 9.76 mm Hg, *p* < 0.05) from baseline after 12 weeks of high-intensity interval training (HIIT). No statistically significant change was observed for the Valsalva ratio, 30:15 ratio, and E:I ratio after 12 weeks of HIIT [38]. Bhati et al. reported no statistically significant change for the E:I ratio (MD 0.0, CI (− 5.59, 5.59), *p* = 0.44), in change in heart rate with standing (MD 3.40, CI (1.51, 5.29), *p* = 0.81), Valsalva ratio (MD 0.36, CI (0.10, 0.62), *p* = 0.47), and change in diastolic blood pressure with handgrip (MD 0.22, CI (− 3.40, 3.84), *p* = 0.77) between intervention and control groups. At the same time, the 30:15 ratio (MD 0.24, CI (− 0.01, 0.49), *p* < 0.001) showed a statistically significant improvement after 12 weeks of resistance training [39].

### Method of measuring cardiac autonomic function

The study by Bhati et al. reported the time of testing of the cardiac autonomic reflex tests [39]. Sacre et al. and Bhati et al. said the cessation of antihypertensive and autonomic nervous system modifying drugs 24 h, respectively, before the testing [35, 39]. One study reported the abstinence from caffeine, alcohol, heavy exercise, and environmental considerations [35]. All three studies reported both groups’ E:I ratio, Valsalva ratio, and 30:15 ratio before and after exercise intervention [35, 38, 39]. Only Bhati et al. reported all parameters of cardiac autonomic reflex tests before and after the exercise intervention [39]. The details of the measurement of cardiac autonomic function are provided in Table 2.

### Meta-analysis on the effect of exercise on E:I ratio

**Fig. 2:**
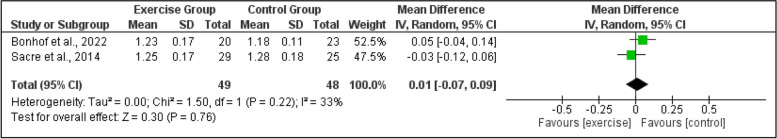
Effect of exercise training on E:I ratio

The three studies measured the E:I ratio [35, 38, 39]. Two studies reported the E:I ratio after 12 weeks of intervention [38, 39]. Exercise training did not have any effect on the E:I ratio (Fig. 2) (mean difference (MD) 0.01, 95% CI − 0.07 to 0.09, participants = 48; *I*^2^ = 33%).

### Meta-analysis on the effect of exercise on Valsalva ratio

**Fig. 3:**
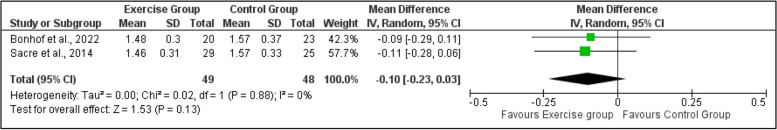
Effect of exercise training on Valsalva ratio

The three studies measured the Valsalva ratio [35, 38, 39]. Two studies reported the Valsalva ratio after 12 weeks of intervention [38, 39]. Exercise training had a significant effect on the Valsalva ratio (Fig. 3) (MD − 0.10, 95% CI − 0.23 to 0.03, participants = 48; *I*^2^ = 0%).

### Meta-analysis on the effect of exercise on 30:15 ratio

**Fig. 4:**
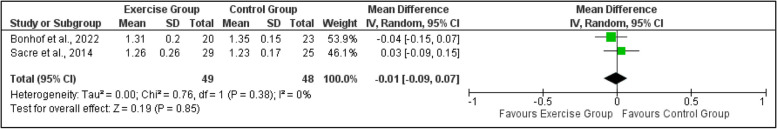
Effect of exercise training on 30:15 ratio

The three studies measured the 30:15 ratio [35, 38, 39]. Two studies reported 30:15 ratio after 12 weeks of intervention [38, 39]. Exercise training did not have any effect on the E:I ratio (Fig. 4) (MD − 0.01, 95% CI − 0.09 to 0.07, participants = 48; *I*^2^ = 0%).

### GRADE assessment

The GRADE Assessment showed a low certainty of the evidence for the outcomes of the E:I ratio and 30:15 ratio and moderate certainty for the Valsalva ratio from two studies [35, 38]. The certainty of evidence was analyzed for the study by Bhati et al. [39], which showed a low certainty of evidence for the three outcomes (E:I ratio, Valsalva ratio, 30:15 ratio). This implies that further evidence is likely to have an important impact on the confidence intervals in the present study in estimating the effect; the estimate is likely to change. The summary of findings is shown in Table 4.

**Table 4:** Summary of findings table—GRADE assessment. Question: What is the effect of exercise training on cardiac autonomic function (cardiac autonomic reflex test variables) in type 2 diabetes mellitus?

Certainty assessment							№ of patients		Effect	Certainty	Importance
**No. of studies**	**Study design**	**Risk of bias**	**Inconsistency**	**Indirectness**	**Imprecision**	**Other considerations**	**Exercise**	**Control**	**Absolute** **(95% CI)**		
**E:I ratio (assessed with cardiac autonomic reflex tests)**											
2	Non-randomized studies	Serious^a^	Serious^b^	Not serious	Serious^c^	All plausible residual confounding would reduce the demonstrated effect	49	48	MD **0.01 higher** (0.07 lower to 0.09 higher)	⨁⨁◯◯ Low	Important
**Valsalva ratio (assessed with cardiac autonomic reflex tests)**											
2	Non-randomized studies	Serious^a^	Not serious	Not serious	Serious^c,d^	All plausible residual confounding would reduce the demonstrated effect	49	48	MD **0.1 lower** (0.23 lower to 0.03 higher)	⨁⨁⨁◯ Moderate	Important
**30:15 ratio (assessed with cardiac autonomic reflex tests)**											
2	non-randomized studies	Serious^a^	Serious^b^	Not serious	Serious^c,d^	All plausible residual confounding would reduce the demonstrated effect	49	48	MD **0.01 lower** (0.09 lower to 0.07 higher)	⨁⨁◯◯ Low	Important
**E: I ratio (follow-up: mean 12 weeks; assessed with: cardiac autonomic reflex tests)**											
1	Randomized trial	Not serious	Not serious	Serious^e^	Serious^e^	None	28	28	MD **0** (− 5.59 lower to 5.59 higher)	⨁⨁◯◯ Low^e,f^	Important
**Valsalva ratio (follow-up: mean 12 weeks; assessed with cardiac autonomic reflex tests)**											
1	Randomized trial	Not serious	Not serious	Serious^e^	Serious^e^	None	28	28	MD **0.36 higher** (0.1 higher to 0.62 higher)	⨁⨁◯◯ Low^e,f^	Important
**30:15 ratio (follow-up: mean 12 weeks; assessed with cardiac autonomic reflex tests)**											
1	Randomized trial	Not serious	Not serious	Serious^e^	Serious^e^	None	28	28	MD **0.24 higher** (0.01 lower to 0.49 higher)	⨁⨁◯◯ Low^e,f^	Important

*CI *Confidence interval, *MD *Mean difference

^a^The studies are non-randomized controlled trials. A likely bias due to confounding and selection bias may be suspected

^b^The intervention in both studies was different, which may have influenced the value

^c^Imprecision may be suspected as there are only two studies included in the analysis

^d^Imprecision may be suspected as there is a wide confidence interval

^e^The small sample cannot represent the population as a whole

^f^Small sample size

## Discussion

The present systematic review showed that exercise training significantly influenced the Valsalva ratio, but not the E:I ratio and 30:15 ratio based on a low to moderate certainty of evidence. This is the first meta-analysis considering the cardiac autonomic function in terms of the cardiac autonomic reflex test outcomes in type 2 diabetes mellitus.

### Exercise intervention

In the present systematic review, a combined aerobic and resistance training program [35], high-intensity interval training [38], and a resistance training program was utilized [39]. Previous systematic reviews reported improved cardiac autonomic function with combined aerobic and resistance training or only an aerobic training program in T2DM [28, 40].

The autonomic nervous system mediates elevated cardiovascular responses and metabolic demands during high-intensity exercise. Previous studies indicated that the long-term modifications of the autonomic nervous system were driven by the dynamic interplay between the feedforward and feedback circuits of the central command and exercise pressor reflex. As these demands are intensity-dependent, HIIT might have promoted superior acute stress [41, 42]. Resistance training increases cardiac vagal activity and reduces sympathetic activity by affecting the baroreflex-NO axis [39]. The pooled analysis showed no effect on the E:I ratio and 30:15 ratio, which could be related to the variable impact of the different exercise interventions in the included studies. The mean difference observed for the E:I ratio in the pooled analysis for the Bonhof et al. study is higher than that observed in the study by Sacre et al. (Fig. 1). This difference is probably due to the higher effect of HIIT on respiratory parameters [43]. The heterogeneity of the treatment intervention needs to be considered for the pooled analysis. Further high-quality randomized controlled trials are needed to assess which type of intervention is needed to demonstrate an improvement in the CARTs outcomes in T2DM.

### Effect of exercise on E:I ratio

Exercise training did not affect the E:I ratio in the current systematic review. The E:I ratio is obtained from the deep breathing test. It is defined as the ratio between the six maximum RR intervals during expiration and six minimum RR intervals during inspiration. It depends on patient compliance, age, respiratory pattern, body position, and baseline and shifting heart rate [24, 44].

The respiratory pattern influences the deep breathing test. T2DM and lung function are closely linked [45]. This reduced lung function in T2DM may be related to microangiopathy of the alveolar capillaries and pulmonary arterioles, chronic low-grade inflammation, and autonomic neuropathy due to loss of elastic recoil of lung parenchyma [46]. These potential mechanisms may affect lung expansion and breathing patterns along with a superimposition of other comorbidities, such as obesity, affecting the E:I ratio. There is a positive correlation between the volume of air inspired during the deep breathing test and the variability in heart rate changes in individuals with diabetes [24]. These changes may have influenced the E:I ratio change in the included studies.

In the study by Bonhof et al., the participants included were overweight adults. High BMI and overweight reduce lung expansion and attenuate chest reflexes due to the influence of mechanical factors such as intrathoracic fat deposits, thereby confounding the E: I ratio [47].

### Effect of exercise on Valsalva ratio

In the present review, exercise training had a significant Valsalva ratio in the exercise group compared to the control group. A study by Vagvolgyi et al. reported a significant improvement in the Valsalva ratio after a 12-week exercise-based intervention in individuals with metabolic syndrome (with and without T2DM). This improvement could be related to increased parasympathetic tone [48]. A previous study reported improved baroreflex sensitivity parameters during the Valsalva maneuver with 4 months of aerobic training in individuals with T2DM [49]. This may be related to the Valsalva maneuver (one of Ewing’s tests), which was reported to have a good sensitivity of about 85% and was a good marker of CAN in T2DM [50] which may have led to a greater effect on the parameter. However, additional high-quality randomized controlled trials are needed to establish the same.

India is the diabetes capital; just one study has addressed the needs of the country in terms of cardiac autonomic function [39, 51]. This study showed a high certainty of the evidence for the three outcomes (E:I ratio, Valsalva ratio, and 30:15 ratio) (Table 4). There is a wide phenotypic variation and prevalence of type 2 diabetes within the country [3]. The study by Bhati et al. was conducted in North India, considering different characteristics of the population, dietary habits, lifestyle [3], and environment [52]. It can be hypothesized that if the same outcome is checked in the other parts of India, it might give a different result considering the characteristics of the population, diet, and lifestyle. Also, the Indian phenotype of T2DM (the Asian Indian Phenotype) is characterized by low BMI, high BF%, higher HbA1C, and associated comorbidities such as obesity and dyslipidemia [53, 54]. These features in the Indian scenario may have led to exercise training having no effect.

### Effect of exercise on 30:15 ratio

There was no effect of exercise on the 30:15 ratio in the present review. 30:15 ratio is defined as the ratio between the longest RR interval between the 25th and the 35th beat and the shortest RR interval between the 10th and the 20th beat in the lying to-standing test [24].

Considering the variable, any changes in RR intervals, such as resting tachycardia, can alter this ratio. The included studies did not have any participants with resting tachycardia. One study reported a normal resting heart rate in exercise and control groups [35]. Thus, it is possible that exercise may not have any effect on the 30:15 ratio of the RR intervals.

The normal 30:15 ratio is ≥ 1.04 per Ewing’s criteria [23]. The 30:15 ratio values in the included studies were normal at baseline, and studies included participants in the early and definite stages of cardiac autonomic neuropathy. As a result, it is anticipated that the 30:15 ratio will not significantly alter after exercise.

Cardiac autonomic reflex tests provide essential information regarding the parasympathetic and sympathetic systems and are considered the gold standard and relevant in the clinical setting [55]. In a busy clinical setting, the performance of all CARTs may not be possible. Previous studies have shown that the deep breathing test, Valsalva maneuver, or the lying to standing test can also be considered for preliminary screening of cardiac autonomic neuropathy in T2DM due to its high sensitivity and specificity [50, 56, 57]. Hence, these tests can be used as a measure to examine the effect of the exercise intervention in T2DM individuals. However, more studies need to explore these outcomes and examine which type of exercise training will be more effective for individuals with T2DM.

## Limitations

The other parameters of cardiac autonomic reflex tests, such as the respiratory sinus arrhythmia ratio, change in systolic blood pressure with standing, and diastolic pressure change with sustained handgrip, could not be considered for the pooled analysis, although a narrative synthesis was performed.

## Conclusion

The present meta-analysis shows that exercise training significantly affects the Valsalva ratio based on a moderate certainty of evidence, with no effect on the 30:15 ratio and E:I ratio considering the low certainty of evidence, with good quality non-RCTs. However, the review highlighted a need for good quality randomized controlled trials utilizing cardiac autonomic reflex tests (which are gold standard and clinically relevant) in individuals with type 2 diabetes mellitus.

## Supplementary Information

Additional file 1. PRISMA checklist.

Additional file 2. Search strategy.

## Data Availability

Not applicable.
