# Supplementary material for: Effect of exercise training on cardiac autonomic function in type 2 diabetes mellitus: a systematic review and meta-analysis
Source: Syst Rev. 2025 Feb 4;14:34. doi: 10.1186/s13643-025-02772-9 (PMC11792330; doi:10.1186/s13643-025-02772-9)
Supplement: Supplementary file 2 — Additional file 2. Search strategy. [file 13643_2025_2772_MOESM2_ESM.docx]

**Supplementary File**

**SEARCH STRATEGY**

**PubMED**

| **Search** | **Query** | **Results** | **Time** |
| --- | --- | --- | --- |
| #4 | Search: **((("type 2 diabetes" OR "Diabetes Mellitus, Type 2"[Mesh] OR "Diabetes Mellitus, Noninsulin-Dependent" OR "Diabetes Mellitus, Non Insulin Dependent" OR "Diabetes Mellitus, Non-Insulin-Dependent" OR "NIDDM" OR "Diabetes Mellitus, Type II" OR "Diabetes mellitus, type 2" OR "Non insulin dependent diabetes mellitus" OR "Diabetes, Type 2" OR (type 2 diabetes with cardiac autonomic neuropathy) OR "cardiac autonomic neuropathy")) AND (("exercis*" OR "exercise"[Mesh] OR "exercise training*" OR "aerobic exercise*" OR "exercise, aerobic" OR "exercise,resistance" OR "resistance exercise*" OR "aerobic training*" OR "resistance training*" OR "physical exercise*" OR "physical activit*" OR "exercise*, physical" OR "acute exercis*" OR "exercis*, acute" OR "isometric exercis*" OR "exercis*, isometric" OR "training, exercise"))) AND (((cardiac autonomic function) OR parasympathetic* OR sympathetic* OR "cardiac autonomic reflex*" OR "cardiac autonomic reflex test*" OR (autonomic function) OR "heart rate variability" OR (cardiac autonomic dysfunction)))** | [368](https://pubmed.ncbi.nlm.nih.gov/?term=%28%28%28%22type+2+diabetes%22+OR+%22Diabetes+Mellitus%2C+Type+2%22%5BMesh%5D+OR+%22Diabetes+Mellitus%2C+Noninsulin-Dependent%22+OR+%22Diabetes+Mellitus%2C+Non+Insulin+Dependent%22+OR+%22Diabetes+Mellitus%2C+Non-Insulin-Dependent%22+OR+%22NIDDM%22+OR+%22Diabetes+Mellitus%2C+Type+II%22+OR+%22Diabetes+mellitus%2C+type+2%22+OR+%22Non+insulin+dependent+diabetes+mellitus%22+OR+%22Diabetes%2C+Type+2%22+OR+%28type+2+diabetes+with+cardiac+autonomic+neuropathy%29+OR+%22cardiac+autonomic+neuropathy%22%29%29+AND+%28%28%22exercis%2A%22+OR+%22exercise%22%5BMesh%5D+OR+%22exercise+training%2A%22+OR+%22aerobic+exercise%2A%22+OR+%22exercise%2C+aerobic%22+OR+%22exercise%2Cresistance%22+OR+%22resistance+exercise%2A%22+OR+%22aerobic+training%2A%22+OR+%22resistance+training%2A%22+OR+%22physical+exercise%2A%22+OR+%22physical+activit%2A%22+OR+%22exercise%2A%2C+physical%22+OR+%22acute+exercis%2A%22+OR+%22exercis%2A%2C+acute%22+OR+%22isometric+exercis%2A%22+OR+%22exercis%2A%2C+isometric%22+OR+%22training%2C+exercise%22%29%29%29+AND+%28%28%28cardiac+autonomic+function%29+OR+parasympathetic%2A+OR+sympathetic%2A+OR+%22cardiac+autonomic+reflex%2A%22+OR+%22cardiac+autonomic+reflex+test%2A%22+OR+%28autonomic+function%29+OR+%22heart+rate+variability%22+OR+%28cardiac+autonomic+dysfunction%29%29%29&size=50&ac=no&sort=relevance) | 05:31:41 |
| #3 | Search:  **((cardiac autonomic function) OR parasympathetic* OR sympathetic* OR "cardiac autonomic reflex*" OR "cardiac autonomic reflex test*" OR (autonomic function) OR "heart rate variability" OR (cardiac autonomic dysfunction))** | [228,115](https://pubmed.ncbi.nlm.nih.gov/?term=%28%28cardiac+autonomic+function%29+OR+parasympathetic%2A+OR+sympathetic%2A+OR+%22cardiac+autonomic+reflex%2A%22+OR+%22cardiac+autonomic+reflex+test%2A%22+OR+%28autonomic+function%29+OR+%22heart+rate+variability%22+OR+%28cardiac+autonomic+dysfunction%29%29&size=50&ac=no&sort=relevance) | 05:31:08 |
| #2 | Search: **("exercis*" OR "exercise"[Mesh] OR "exercise training*" OR "aerobic exercise*" OR "exercise, aerobic" OR "exercise,resistance" OR "resistance exercise*" OR "aerobic training*" OR "resistance training*" OR "physical exercise*" OR "physical activit*" OR "exercise*, physical" OR "acute exercis*" OR "exercis*, acute" OR "isometric exercis*" OR "exercis*, isometric" OR "training, exercise")** | [647,881](https://pubmed.ncbi.nlm.nih.gov/?term=%28%22exercis%2A%22+OR+%22exercise%22%5BMesh%5D+OR+%22exercise+training%2A%22+OR+%22aerobic+exercise%2A%22+OR+%22exercise%2C+aerobic%22+OR+%22exercise%2Cresistance%22+OR+%22resistance+exercise%2A%22+OR+%22aerobic+training%2A%22+OR+%22resistance+training%2A%22+OR+%22physical+exercise%2A%22+OR+%22physical+activit%2A%22+OR+%22exercise%2A%2C+physical%22+OR+%22acute+exercis%2A%22+OR+%22exercis%2A%2C+acute%22+OR+%22isometric+exercis%2A%22+OR+%22exercis%2A%2C+isometric%22+OR+%22training%2C+exercise%22%29&size=50&ac=no&sort=relevance) | 05:30:17 |
| #1 | Search: **("type 2 diabetes" OR "Diabetes Mellitus, Type 2"[Mesh] OR "Diabetes Mellitus, Noninsulin-Dependent" OR "Diabetes Mellitus, Non Insulin Dependent" OR "Diabetes Mellitus, Non-Insulin-Dependent" OR "NIDDM" OR "Diabetes Mellitus, Type II" OR "Diabetes mellitus, type 2" OR "Non insulin dependent diabetes mellitus" OR "Diabetes, Type 2" OR (type 2 diabetes with cardiac autonomic neuropathy) OR "cardiac autonomic neuropathy")** | [234,661](https://pubmed.ncbi.nlm.nih.gov/?term=%28%22type+2+diabetes%22+OR+%22Diabetes+Mellitus%2C+Type+2%22%5BMesh%5D+OR+%22Diabetes+Mellitus%2C+Noninsulin-Dependent%22+OR+%22Diabetes+Mellitus%2C+Non+Insulin+Dependent%22+OR+%22Diabetes+Mellitus%2C+Non-Insulin-Dependent%22+OR+%22NIDDM%22+OR+%22Diabetes+Mellitus%2C+Type+II%22+OR+%22Diabetes+mellitus%2C+type+2%22+OR+%22Non+insulin+dependent+diabetes+mellitus%22+OR++%22Diabetes%2C+Type+2%22+OR+%28type+2+diabetes+with+cardiac+autonomic+neuropathy%29+OR+%22cardiac+autonomic+neuropathy%22%29&size=50&ac=no&sort=relevance) | 05:20:41 |

Showing 1 to 4 of 4 entries

**Ovid Medline**

| [# ▲](https://ovidsp.dc1.ovid.com/ovid-b/ovidweb.cgi?&S=OIJKFPMJIPACLBEDKPLJOEPMIGCIAA00&Sort+Sets=descending) | **Searches** | **Results** | **Type** |
| --- | --- | --- | --- |
| 1 | (type 2 diabetes or diabetes mellitus, non insulin dependent or diabetes mellitus, type 2 or diabetes mellitus, type ii or diabetes, type 2 or niddm or non-insulin-dependent diabetes mellitus or cardiac autonomic neuropathy).mp. | 234466 | Advanced |
| 2 | (cardiac autonomic function or parasympathetic* or sympathetic* or cardiac autonomic reflex test* or cardiac autonomic reflex* or autonomic function* or heart rate variability or cardiac autonomic dysfunction*).mp. | 140840 | Advanced |
| 3 | (exercis* or activit*, physical or acute exercis* or aerobic exercis* or exercis*, acute or exercis*, aerobic or exercis*, isometric or exercis*, physical or exercise training* or isometric exercis* or physical activity or physical exercise* or training, exercise or resistance training* or resistance exercise* or exercis*,resistance or training*, resistance).mp. | 544332 | Advanced |
| 4 | 1 and 2 and 3 | 238 |  |

**EMBASE**

Top of Form

#1 AND #2 AND #3

[275](https://www.embase.com/)

**#3**

**'cardiac autonomic function'**/exp OR **'heart rate variability'**/exp OR **'cardiac autonomic reflex'** OR **'cardiac autonomic dysfunction'**/exp

[34,842](https://www.embase.com/)

**#2**

**'exercise'**/exp OR **'effort'** OR **'exercise capacity'** OR **'exercise performance'** OR **'exercise training'** OR **'exertion'** OR **'fitness training'** OR **'fitness workout'** OR **'physical conditioning, human'** OR **'physical effort'** OR **'physical exercise'** OR **'physical exertion'** OR **'physical work-out'** OR **'physical workout'** OR **'exercise'** OR **'physical activity'**/exp

[1,306,132](https://www.embase.com/)

**#1**

(**'non insulin dependent diabetes mellitus'**/exp OR **'diabetes mellitus type 2'** OR **'diabetes mellitus type ii'** OR **'diabetes mellitus, non insulin dependent'** OR **'diabetes mellitus, non-insulin-dependent'** OR **'diabetes mellitus, type 2'** OR **'diabetes mellitus, type ii'** OR **'diabetes type 2'** OR **'diabetes type ii'**) AND **o** AND **'dm 2'** OR **'niddm'** OR **'niddm (non insulin dependent diabetes mellitus)'** OR **'non insulin dependent diabetes'** OR **'non-insulin-dependent diabetes mellitus'** OR **'noninsulin dependent diabetes'** OR **'noninsulin dependent diabetes mellitus'** OR **'t2dm'** OR **'type 2 diabetes'** OR **'type 2 diabetes mellitus'** OR **'type ii diabetes'** OR **'type ii diabetes mellitus'** OR **'non insulin dependent diabetes mellitus'** OR **'cardiac autonomic neuropathy'**/exp

[395,186](https://www.embase.com/)

Bottom of Form

**CINAHL**

| **Search ID #** | **Search** | **Limiters/expanders** | **Last Run Via** | **Results** |
| --- | --- | --- | --- | --- |
| S3 | TX ( ( "Diabetes Mellitus, Type 2") OR "type 2 diabetes or type 2 diabetes mellitus or t2dm or t2d or niddm or "non-insulin dependent diabetes mellitus" OR "Diabetes Mellitus, Type II" OR "Non insulin dependent diabetes mellitus" OR "Diabetes, Type 2" OR "cardiac autonomic neuropathy") ) AND TX ( exercise or physical activity or fitness or aerobic training or strength training or cardiovascular training or exercise, aerobic" OR "exercise,resistance" OR "physical exercise" "exercise, physical" OR "acute exercise" OR "exercise, acute" OR "isometric exercis*" OR "exercise, isometric" OR "training, exercise" ) AND TX ( cardiac autonomic function or parasympathetic* OR sympathetic* OR "cardiac autonomic reflex*" OR "cardiac autonomic reflex test*" OR (autonomic function) OR "heart rate variability" OR (cardiac autonomic dysfunction) ) | Expanders - Apply equivalent subjects Search modes - Boolean/Phrase | Interface - EBSCOhost Research Databases Search Screen - Advanced Search Database - CINAHL Complete | 276 |
| S2 | TX ( ( "Diabetes Mellitus, Type 2") OR "type 2 diabetes or type 2 diabetes mellitus or t2dm or t2d or niddm or "non-insulin dependent diabetes mellitus" OR "Diabetes Mellitus, Type II" OR "Non insulin dependent diabetes mellitus" OR "Diabetes, Type 2" OR "cardiac autonomic neuropathy") ) AND TX ( exercise or physical activity or fitness or aerobic training or strength training or cardiovascular training or exercise, aerobic" OR "exercise,resistance" "physical exercise" "exercise, physical" OR "acute exercise" OR "exercise, acute" OR "isometric exercis*" OR "exercise, isometric" OR "training, exercise" ) | Expanders - Apply equivalent subjects Search modes - Boolean/Phrase | Interface - EBSCOhost Research Databases Search Screen - Advanced Search Database - CINAHL Complete | 11,116 |
| S1 | TX ( "Diabetes Mellitus, Type 2") OR "type 2 diabetes or type 2 diabetes mellitus or t2dm or t2d or niddm or "non-insulin dependent diabetes mellitus" OR "Diabetes Mellitus, Type II" OR "Non insulin dependent diabetes mellitus" OR "Diabetes, Type 2" OR "cardiac autonomic neuropathy") | Expanders - Apply equivalent subjects Search modes - Boolean/Phrase | Interface - EBSCOhost Research Databases Search Screen - Advanced Search Database - CINAHL Complete | 71,329 |

**Web of Science**

| **Search no.** |  | **Results** |
| --- | --- | --- |
| **4** | **#1 AND #2 AND #3** | [363](https://www.webofscience.com/wos/woscc/summary/76e6c9fd-8f8c-452b-8ef4-6c18231c7aa2-9fcfd8b6/relevance/1) |
| **3** | **ALL=((cardiac autonomic function) OR parasympathetic* OR sympathetic* OR "cardiac autonomic reflex*" OR "cardiac autonomic reflex test*" OR (autonomic function) OR "heart rate variability" OR (cardiac autonomic dysfunction))** | [142,423](https://www.webofscience.com/wos/woscc/summary/5b9169fa-cbf5-424e-869c-dd1bcbea9a43-9fcfd73e/relevance/1) |
| **2** | **ALL=("exercis*" OR "exercise training*" OR "aerobic exercise*" OR "exercise, aerobic" OR "exercise,resistance" OR "resistance exercise*" OR "aerobic training*" OR "resistance training*" OR "physical exercise*" OR "physical activit*" OR "exercise*, physical" OR "acute exercis*" OR "exercis*, acute" OR "isometric exercis*" OR "exercis*, isometric" OR "training, exercise")** | [766,361](https://www.webofscience.com/wos/woscc/summary/5376299c-49a6-4594-a640-ca141dd432bf-9fcfd13b/relevance/1) |
| **1** | **ALL=(("type 2 diabetes" OR "Diabetes Mellitus, Noninsulin-Dependent" OR "Diabetes Mellitus, Non Insulin Dependent" OR "Diabetes Mellitus, Non-Insulin-Dependent" OR "NIDDM" OR "Diabetes Mellitus, Type II" OR "Diabetes mellitus, type 2" OR "Non insulin dependent diabetes mellitus" OR "Diabetes, Type" OR "cardiac autonomic neuropathy"))** | [211,543](https://www.webofscience.com/wos/woscc/summary/4dd7b00f-1888-425a-b588-ab8599317df5-9fcfcbe0/relevance/1) |

**SCOPUS**

( TITLE-ABS-KEY ( type 2 diabetes ) AND TITLE-ABS-KEY ( cardiac AND autonomic AND function ) AND TITLE-ABS-KEY ( exercise ) ) AND ( EXCLUDE ( EXACTKEYWORD , "Animals" ) OR EXCLUDE ( EXACTKEYWORD , "Animal" ) ) AND ( EXCLUDE ( DOCTYPE , "ch" ) OR EXCLUDE ( DOCTYPE , "ed" ) OR EXCLUDE ( DOCTYPE , "le" ) )

**75**
